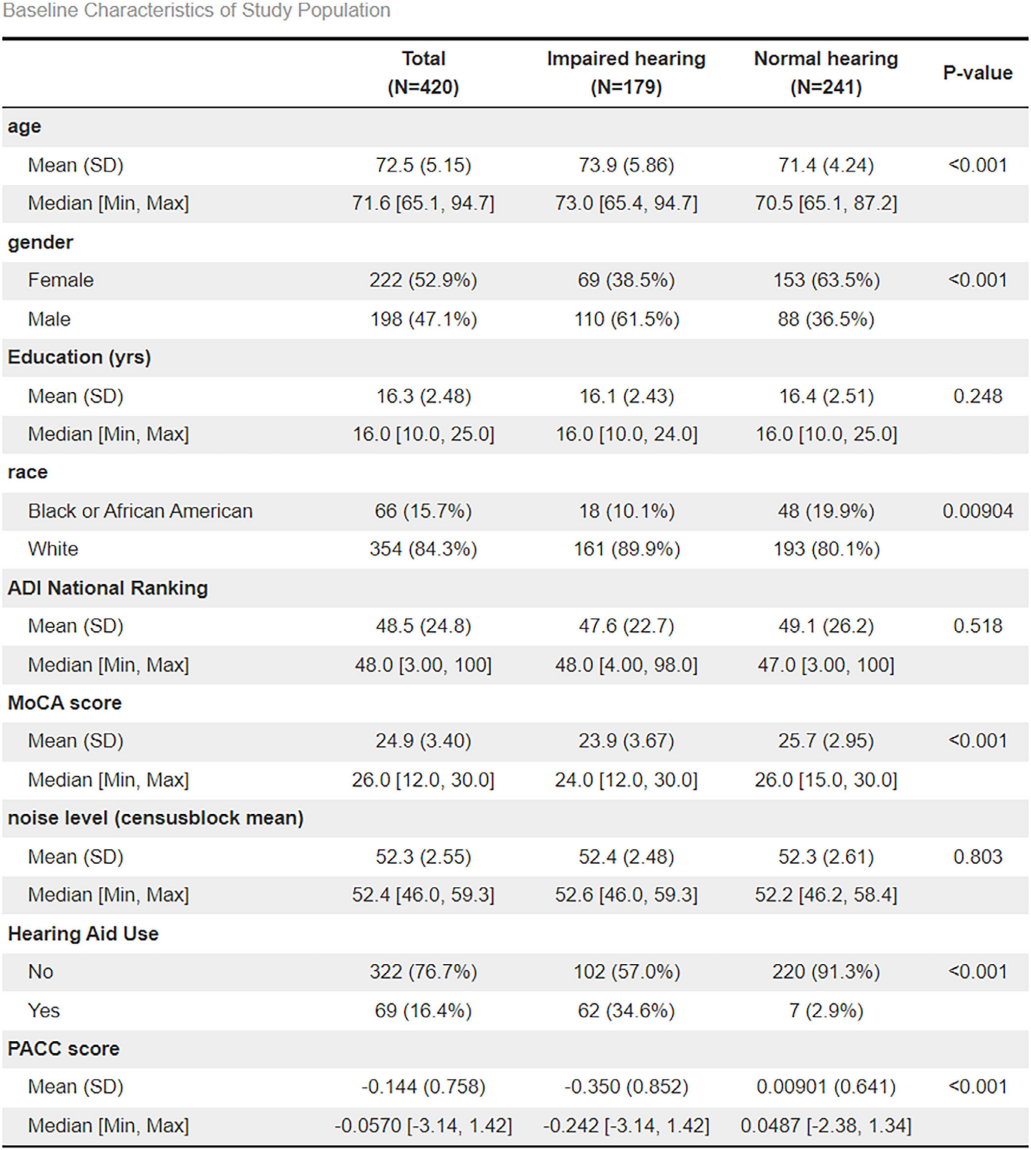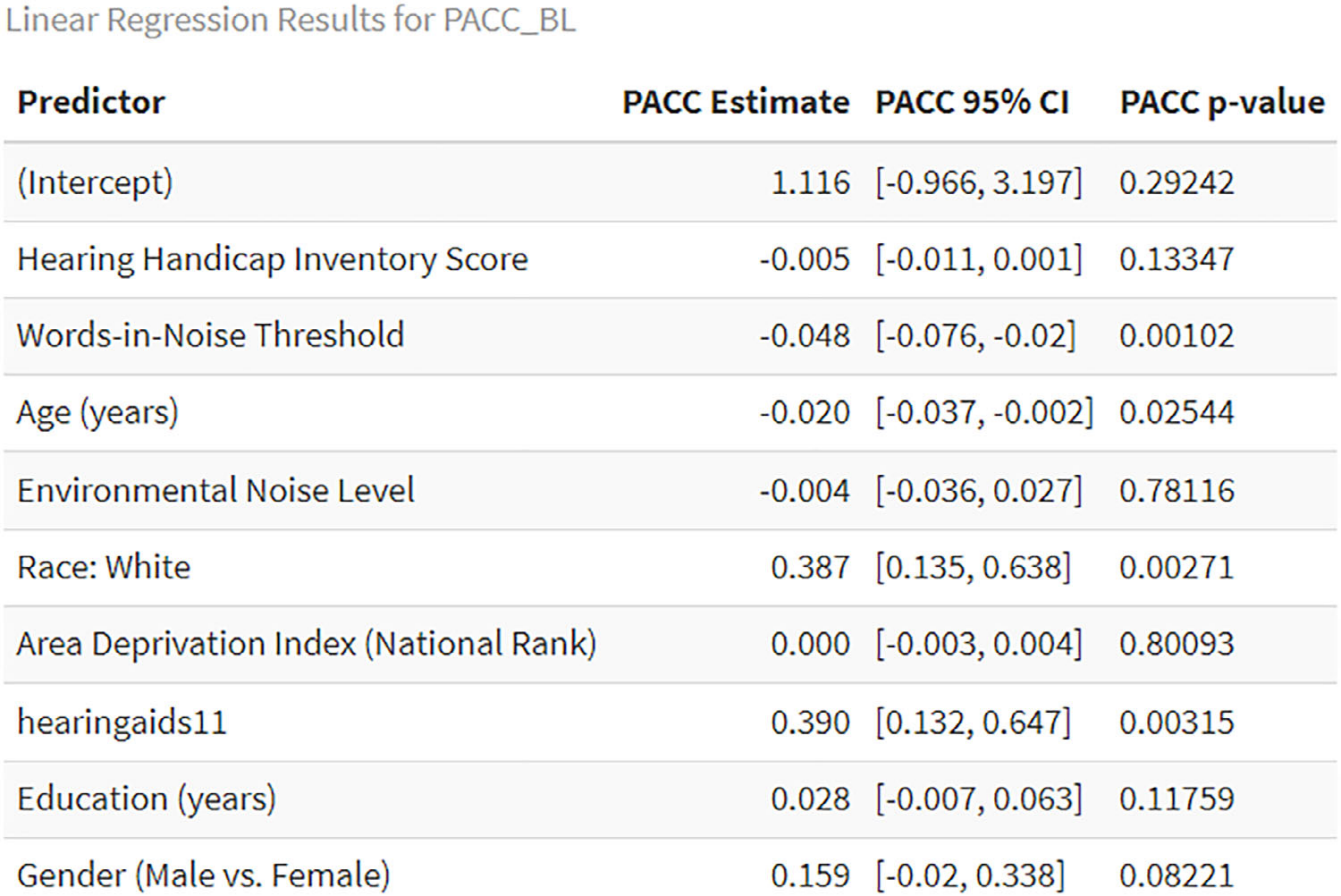# Hearing Impairment, Hearing Aid Use, and Cognitive Outcomes in Older Adults: *A Cross‐Sectional Study*

**DOI:** 10.1002/alz70860_106643

**Published:** 2025-12-23

**Authors:** Semere Bekena, Ramkrishna Kumar Singh, Yiqi Zhu, Nikitha Damera, Kaylin Taylor, Jean‐Francois Trani, Ganesh M. Babulal

**Affiliations:** ^1^ Washington University School of Medicine, Saint Louis, MO, USA; ^2^ Washington University School of Medicine, St. Louis, MO, USA; ^3^ Washington University, St. Louis, MO, USA; ^4^ Natiobal conservatory of Arts and Crafts, Paris, NA, France; ^5^ University of Johannesburg, Johannesburg, Gauteng Province, South Africa; ^6^ Knight Alzheimer Disease Research Center, St. Louis, MO, USA

## Abstract

**Background:**

The 2024 Lancet Commission on Dementia Prevention recognizes midlife hearing loss as a modifiable contributor to dementia risk. Hearing impairment affects approximately 68% of U.S. adults over 70 and is associated with accelerated cognitive aging, with a 25 dB hearing deficit linked to cognitive decline equivalent to 6.8 years of aging. This study assessed hearing impairment, measured via NIH Toolbox Words‐in‐Noise (WIN), and its influence on cognition while adjusting for area deprivation index (ADI), race, neighborhood noise levels, and self‐perceived hearing difficulty using the Hearing Handicap Inventory (HHI). Secondary analysis examined if associations differed by race and hearing aid use.

**Method:**

We conducted a cross‐sectional study of 420 older adults (mean age 72.5 ± 5.15 years) classified as having impaired (*n* = 179) or normal hearing (*n* = 241) based on WIN thresholds. Data on hearing aid use, HHI scores, demographics, neighborhood noise, and ADI were collected. Cognitive function was assessed using the Montreal Cognitive Assessment (MoCA) and Preclinical Alzheimer Cognitive Composite (PACC). Multiple linear regressions examined associations between hearing measures and cognition, adjusting for covariates. Interaction terms for hearing, race, and hearing aid use were tested. Statistical significance was set at *p* <0.05.

**Result:**

Participants with impaired hearing (*n* = 179) were older (73.9 vs. 71.4 years, *p* <0.001), often male (61.5% vs. 36.5%, *p* <0.001), and had lower cognitive scores than normal‐hearing individuals (*n* = 241). Both PACC and MoCA assessments showed similar results, with impaired‐hearing participants performing worse on PACC (‐0.35 vs. 0.009, *p* <0.001) and MoCA (23.9 vs. 25.7, *p* <0.001). Race distributions differed (*p* = 0.009), but neighborhood‐level deprivation (*p* = 0.518) did not. In adjusted models, higher (worse) WIN thresholds were significantly associated with lower cognitive scores (*p* <0.01) in MoCA and PACC, controlling for age, sex, race, education, and neighborhood noise levels. Hearing aid use was associated with higher PACC scores (*p* = 0.003), suggesting a protective effect on cognitive function. However, HHI scores were still not significantly related to cognitive outcomes. All interaction terms were not significant.

**Conclusion:**

Hearing impairment is significantly associated with lower cognitive performance, while hearing aid use may provide cognitive benefits. However, self‐reported hearing difficulty (HHI) was not a significant predictor of cognition, highlighting the importance of objective hearing assessments.